# Predicting the cost of COVID-19 treatment and its drivers in Indonesia: analysis of claims data of COVID-19 in 2020-2021

**DOI:** 10.1186/s13561-022-00392-w

**Published:** 2022-08-31

**Authors:** Ryan R. Nugraha, Mutia A. Pratiwi, Ruli Endepe Al-Faizin, Ardian Budi Permana, Ery Setiawan, Yuli Farianty, Kalsum Komaryani, Hasbullah Thabrany

**Affiliations:** 1 USAID Health Financing Activity/ThinkWell, LLC, Plaza Bank Index, Level 11, Jalan M.H. Thamrin No.57, RW.5, Gondangdia, Kec. Menteng, Central Jakarta, 10350 Indonesia; 2 Center of Health Financing and Decentralization Policy, Ministry of Health, Jalan Percetakan Negara No.29, Rawasari, Kec. Johar Baru, Central Jakarta, Indonesia

**Keywords:** Claim payment, Retrospective payment, Medical cost, COVID-19, Indonesia

## Abstract

**Background:**

Recent Coronavirus Disease-19 (COVID-19) pandemic shows that health system, particularly hospital care, takes the highest toll on COVID-19. As hospital gets to manage the surge of COVID-19 cases, it is important to standardize treatment standard and package for COVID-19. Until recently, in Indonesia, COVID-19 curative package in hospital is paid using a retrospective payment system (claims system) using a per-diem rate. Quantifying standard cost using an established retrospective claims dataset is important as a basis for standard formulation for COVID-19 package treatment, should COVID-19 be accommodated into the benefit package for Universal Health Coverage (UHC) under the National Health Insurance.

**Methods:**

We estimated a standard cost for COVID-19 treatment using provider’s perspective. The analysis was conducted retrospectively using established national COVID-19 claims dataset during January 2020 until 2021. Utilizing individual-or-patient level analysis, claims profile were broken down per-patient, yielding descriptive clinical and care-related profile. Estimate of price and charge were measured in average. Moreover, indicators were regressed to the total charged price (in logarithmic scale) so as to find the predictors of cost.

**Results:**

Based on the analysis of 102,065 total claims data received by MOH in 2020-2021, there is an average claim payment for COVID-19 in the amount of IDR 74,52 million (USD$ 5175). Significant difference exists in hospital tariffs or price to the existing claims data, indicating profit for hospital within its role in managing COVID-19 cases. Claim amount predictors were found to be associated with change of claim amount, including high level of severity, hospital class, intensive care room occupancy and ventilator usage, as well as mortality.

**Conclusion:**

As COVID-19 pandemic shifts towards an endemic, countries including Indonesia need to reflect on the existing payment system and move towards a more sustainable payment mechanism for COVID-19. The COVID-19 payment system needs to be integrated into the existing national health insurance allowing bundled payment to become more sustainable, which can be achieved by comprehensively formulating the bundled payment package for COVID-19.

## Introduction

COVID-19, having started as an outbreak initially emerging in Wuhan, People’s Republic of China during the last month of 2019, had prompted countries to respond to what turned out to be global pandemic. Numerous countries, namely, South Korea, Taiwan, and Vietnam, made swift responses in order to contain the spread, as the disease started to aggressively infect its citizens, and Indonesia is no exception to this. On March 2nd, 2020, the government of Indonesia (GoI) officially confirmed its first cases and a couple of weeks later, WHO declared COVID-19 as a pandemic. As of March 8th of 2022, Indonesia recorded 5,770,105 confirmed cases with over 150,430 deaths [1]. The recent peak of case surge was felt ultimately due to the spread of the Omicron variant over the early months of the year 2022.

The health system was deeply affected by the rise of COVID-19, particularly as the capacity of our health system influences COVID-19 outcome [2]. As part of health system, ultimately, hospitals take the highest toll of burden due to COVID-19. According to the unpublished data from the Ministry of Health, almost as many as 970 hospitals are assigned as a special hospital for COVID-19, from a total of 2996 operating hospitals in the nation [3]. Hospitals were receiving overwhelming cases of COVID-19 since they were incorporated in the COVID system, with its peak during the first week of January and June 2021. The latter is arguably because of the Delta variant spread.

Financing in hospital-level care becomes imperative to sustain healthcare services, particularly for COVID-19 patients. Sustaining hospital care is even more important as the pursuit for universal health coverage [4]. As the number of cases is increasing, especially the number of cases needing treatment, financing channels need to be established so as to not impose a financial burden on patients. Fortunately, Law No 6 Year 2018 on Health Quarantine (“Law No 6 Year 2018”) facilitates the channeling of funding using the national budget, particularly for COVID-19 case management, as COVID-19 has been regarded as a pandemic. Within Law No 6 Year 2018 on Health Quarantine, it is further explained that coverage includes upstream population-based or public health services such as tracing program and downstream approaches such as ensuring equal curative services at both the primary to tertiary level.

As COVID-19 care financial coverage is to be supported by the national budget, the Ministry of Health (MOH) decided to establish a special payment scheme for accommodating the secondary level of care for COVID-19 in 2020. This special payment scheme is based on a retrospective payment scheme using a per-diem rate (daily rate), accommodating care packages for COVID-19 (or “claim” payment scheme). The MOH established a claim application system, in which hospitals can submit COVID-19 claims or charge based on services provided, using a pre-established daily rate. The Social Security Agency for Health (SSAH*)* was assigned as the verifier of the submitted claims or charges to the system, while the payment is incurred by the MOH. The overall regulation was incorporated into the regulation of the Decree of Minister of Health No 446 Year 2020, which was revised by Decree of Minister of Health No 4344 Year 2021 [5, 6]. The payment scheme, along with its application system, is already adjusted to the growing practice of COVID-19 case management; however, a standard of cost needs to be established to improve efficiency. Despite the standard rate applied along with the supportive claim system, there is wide variance within the claims attributed to several factors, which may harm the sustainability of COVID-19 service in the long run [7]. Given this backdrop of financial coverage at a national level, this study asks: what are the existing associated costs and drivers of these costs of Covid-19 treatment, and in this respect, how can the standard cost be predicted using this data?

In order to establish a standard protocol for practice as well as a more robust payment scheme, it is essential to approximate the standard cost using updated evidence. In this paper, we argue that by analyzing tariffs and claims using COVID-19 claims within the MOH level – both in individual unit analysis and costs incurred during COVID-19 treatment at the hospital level, the standard cost of Covid-19 can be predicted.

The following paragraphs firstly describe method-wise data sources and the study population for this study. Results and study findings are in the second section, with which claims data are scrutinized with hospital class, clinical aspects of care and severity level as the categories used. The third section looks at the discussion which arises from the key findings of the study. The last section provides summary and policy recommendations.

## Methods

### Data sources and variables

This study is a retrospective cross-sectional study which utilizes MOH Indonesia’s existing COVID-19 e-claim billing database collected from January 2020 until January 2021. A total sample of 102,065 patients was submitted for analysis. The claims can only be processed if they suit the COVID-19 criterion of having been confirmed with Polymerase Chain Reaction (PCR) test. Any hospital, regardless of class, which is proficient in providing COVID-19 services was able to submit reimbursement claims. Within the claims database, COVID-19 is defined using the International Classification of Diseases (ICD)-10 diagnostic code of “Coronavirus Infection, Unspecified Site” (code B34.2).

Within the claims application, data points such as clinical and charge or claims profiles were available for analysis. Medical charges were divided into several sub-components, including drugs and consumables, healthcare workers fee, examinations, as well as room charges, as it becomes the cost component which shapes COVID-19 care at the hospital level. As an individual analysis-unit data, clinical profile, as well as healthcare-seeking were recorded and incorporated into analysis, including COVID-19 status, hospital-related indicators, and others.

### Model structure and data analysis

The claims data were matched with clinical profile data within claims applications with a purpose to lay out a descriptive profile of COVID-19 patients. In clinical terms, the average length-of-stay for COVID-19 patients was calculated within the period, as well as a proportion of COVID-19 cases. Hospital-related indicators such as hospital class and intensive care unit (ICU) occupancy, among others, were analyzed using descriptive statistics, and were also used in regression as covariates to predict COVID19 costs. The claims data used for this analysis were in 2020-2021. Data was collected by the lead author in February 2021. Data analysis by authors was carried out in May-June 2021.

**Fig. 1:**
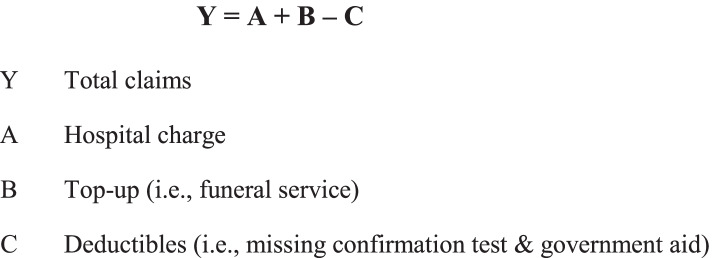
Claims Formula

Claims charges were analyzed and compared with average hospital tariffs to find out the difference in cost. The claims were also analyzed based on disease severity to find the difference in cost predictors among different disease categories, using a student t-test. In descriptive terms, the claims were calculated using the following formula (Fig. 1):

Submitted hospital charge (*A*) was adjusted to several factors, including length of stay (LOS)^1^. After hospital charges were concluded, it is added with any cost incurred as a top-up (or addition, *B*), such as the cost for funeral service (which was also covered within national budget). Subsequently, it was subtracted with deductibles (C), such as punishment for a missing confirmation test. This calculation will yield total claims (Y).

After performing descriptive univariate and bivariate analysis, a subsequent t-test was performed to see if there was any difference in regard to submitted charges or claims between subgroups, particularly based on different disease severities.

**Fig. 2:**
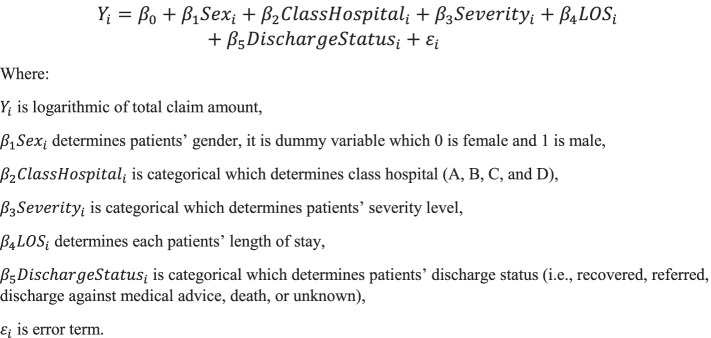
Regression Model or Equation

Next, the study employed multi-variable regression analysis to find out predictors as well as its correlation with total claims amount. The model is presented in Fig. 2.

The claims data was calculated for regression analysis using weighted regression with logarithmic claims amount as dependent variable; the data was regressed for multiple variables, including severity, hospital classes, use of ventilator, occupancy of intensive care unit, as well as outcome. This method is carried out since there would be potential heteroskedasticity if applied using Ordinary Least Squares (OLS) regression. The regression model was developed by using weighted OLS or weighted least squares (WLS) in order to address the heteroskedasticity issues which could occur if simple OLS regression were to be used. The equation function is developed to match the linear regression method employed in the analysis. The dependent variable was, in fact, the actual amount of claim reimbursement that was observed by the lead author. The independent variables were selected from the variables indicated to have driven the amount of reimbursement.

The logarithmic regression was used to predict percent of change of claim amount per addition of unit within independent variables. The regression uses *p*-value significant to the level of 0.01.

## Results

The study observed the profile of 102,065 COVID-19 patients based on the admitted claims data. These claims data were gathered from all hospitals in Indonesia, particularly those that have isolation rooms and ICU as minimum standards for COVID-19 patients.

There was little variation at the individual level of COVID-19 patients based on submitted claims data. The gender distribution of patients was quite balanced between males (51.9%) and females (48.1%). Based on COVID-19 status, 68.02% of sample patients were confirmed patients, while suspected patients make up 20.73%, while probable and person under surveillance comprises 11.25% of total patients.^2^ As it happens, there is an existing study on the association between COVID-19 and gender was conducted by Gebhard et al. in 2020, where they found that a higher fatality rate was higher among male patients compared to female counterparts. Notably, this finding was not evident in our study.

Of all patients admitted, not all patients were intentionally treated for COVID-19; instead, there were patients caught having been infected by COVID-19 while being at a hospital (acquired infection) (Table 1).

**Table 1:** Patients’ characteristic based on submitted claims

Variable	n	Proportion
***N*** **= 102,065**		
**Sex**		
Female	49,340	48.1%
Male	53,265	51.9%
**Hospital Class**		
A	11,859	11.56%
B	42,292	41.22%
C	42,058	40.99%
D	6396	6.23%
**Covid-19 Status (Upon Entry)**		
Probable	5779	5.63%
Person(s) under Surveillance (ODP)	5760	5.62%
Suspect	21,272	20.73%
Confirmed	69,794	68.02%
**Severity**		
Mild	63,631	62.0%
Moderate	17,652	17.2%
Severe or Critical	21,322	20.8%
**Discharge Status**		
Fully recovered	79,461	77.44%
Referred	5056	4.93%
Discharge against Medical Advice (DAMA)	3163	3.08%
Died	13,820	13.47%
Other	1105	1.08%
**ICU Admission**		
No ICU	92,734	90.38%
ICU	9871	9.62%

Claims data is essential in informing clinical care. Of all patients’ data submitted for claims, 77.44% were recovered, with 13.47% reported died, 3.08% were discharged against medical advice (DAMA), while up to 5% was referred to another hospital. However, at the early days of the pandemic, many hospitals were burdened by a high influx of patients; thus, the mortality rate might not be reflecting the valid rate as people admitted to hospital were relatively in critical condition.

Prior to going to the findings, it is warranted to elucidate as regards classification of hospitals which is regulated in the Ministry of Health’s decree No. 3 Year 2020. This regulation stipulates the following classifications: (i) Class A hospitals are hospitals which have at least 250 hospital beds; (ii) Class B hospitals are hospitals which have at least 200 hospital beds; (iii) Class C hospitals are hospitals which have at least 100 hospital beds; (iv) Class D hospitals are hospitals which have at least 50 hospital beds. At the top of the pyramid, Class A hospitals have a higher number of specialists, and medical equipments as they are at the most advanced level of the referral system. In terms of treatments of COVID-19, Class A hospitals tend to give complex treatments to patients hence higher costs which result in higher charges imposed in treating COVID-19 patients.

The majority of patients visited Class B and C hospitals, which were distributed across Indonesia and were proficient in providing COVID-19 services. Such hospitals were mandated by the provincial government to become COVID-19 treatment centers in each respective province or region. Almost 41% came to a Class-C hospital while 41.2% came to a Class-B Hospital. Only 11.56% came to a Class-A Hospital, which comprised of end-level referral public and teaching hospitals located across Indonesia, centered around provincial and national capitals. Only 6.23% of COVID-19 patients came to a D-level hospital which had little-to-minimal resources and COVID-19 care.

The majority of patients admitted to hospital and paid by the claim mechanism consisted of mild COVID-19 cases. More than half (62%) of the submitted medical claims were patients with mild cases upon arrival. Patients with moderate-level severity made up 17.2% of the claims, while 20.8% of the total were categorized as either having severe or critical COVID-19 symptoms. These ratios were, in fact, not consistent with regulation which allows only moderate COVID-19 severity to be admitted. Moreover, further clarification is needed to acknowledge if patients admitted with mild cases of COVID-19 have associated comorbidities (Table 2).

**Table 2:** Length of stay of COVID-19 patients

	Mean (days)	SD
**Length of Stay (LOS)**	8.90	± 5.88
LOS in ICU	0.53	± 2.28

Length-of-stay of COVID-19 patients would need particular attention, especially in regard to severity of COVID-19 cases, costs and hospitals. More severe COVID-19 patients would require particular clinical treatments and medicines. Thus, patients with higher severity levels take more time to be treated in hospital compared to other COVID-19 patients with milder symptoms. In addition, longer LOS would consequently drive higher treatment costs for patients with higher severity levels due to specific clinical treatments and medicines given during treatment. Other cost components, namely, doctors’ visits, room charges, and medical consumables, are higher if LOS are longer.

Claims data is also beneficial to shed light on evidence related to clinical aspects of care, particularly for length-of-stay (LOS). On average, patients were admitted for 8-9 days (length of stay/LOS), while it was just a little more than a day for ICU inpatient stays. Ultimately, LOS becomes an important indicator for rationalizing tariffs and adequacy of rate payment.

The breakdown of medical costs depicted in Table 3 portrays costs from the perspective of the hospital. Room charges were the most expensive component of COVID-19 tariffs, with an average of total 7.42 million (USD 515) allotted, comprising of isolation room (IDR 6,7 million; USD 471) and ICU top-up (IDR 634.780; USD 44). A significant charge evident from the data is attributed to the fact that COVID-19 inpatient rooms are required to be under negative pressure.

**Table 3:** COVID-19 hospital tariff breakdown

***N*** = 102.605		
Components^**a**^	Average^**b**^	SD
**Medical Procedures**	**2839**	**13,616.15**
*Non-Surgical Procedures*	2427.92	13,345.05
*Surgical Procedures*	311,84	2527.01
*Blood Transfusion*	83,91	768.13
*Rehabilitation*	15,77	455.29
**Healthcare Workers’ Fee**	**4731.06**	**11,407.14**
*Doctors’ visit*	2109.6	6192.04
*Expert consultation (*i.e.*, specialist)*	209.11	2043.59
*Nursing fee*	2412.34	9205.66
**Tests & Examinations**	**3862.21**	**6266.31**
*Supporting Examination*	480.58	2716.18
*Radiologic Exam*	498.70	1133.375
*Laboratorium Exam*	2882.92	4657.25
**Accommodations**	**7422.04**	**13,786.09**
*Isolation Room*	6787.27	12,819.96
*Intensive Care Unit (ICU)*	634.78	4954.45
**Medical Devices**	**2676.35**	**9708.08**
*Medical Devices*	2134.16	8431.70
*Rented Devices*	542.19	3140.50
**Consumables**	**2776.98**	**8111.84**
**Drugs**	**5789.30**	**15,100.04**
*Regular Medicines*	5772.55	15,091.28
*Chronic & Other Drugs*	16.75	450.28

^a^Costs were incurred per-diem (daily)

^b^All in,000 IDR (thousands, Indonesian Rupiah), with conversion rate of 1US$ = IDR 14,400

Reported charges for medical drugs were deemed as the second most expensive charge for COVID-19 treatment. Drugs’ tariff allotted approximately IDR 5789 million (USD 402), which pervaded into regular medicines for COVID-19 (IDR 5772,000, USD 400.83) and chronic drugs (IDR 16,750; USD 1.16). COVID-19 drug treatment absorbed a huge proportion of cost as COVID-19 treatment package is yet to be standardized. Moreover, the claim variable of chemotherapy and chronic drugs was particularly set to accommodate the usage of drugs to treat existing comorbidities.

As COVID-19 requires close observation, it is worth noting that nurses and doctors require adequate compensation, hence their tariff became the 3rd highest on the claims list. Healthcare workers fee took up an average of IDR 4731,060 (USD 328.5) per patients, with tariff breakdown namely including doctors’ visits (IDR 2109,000; USD 146.5), expert or specialist consultations (IDR 209,110; USD 14.5), as well a as nursing fee (IDR 2412,340; USD 167.52).

The cost component of the claims payment for COVID-19 services is differentiated based on the system itself, where each shows the flow of COVID-19 payments in secondary level care. Ultimately, our analysis yielded that two of the most important components were tariffs, averaging IDR 30,098,590 (USD 2090) and total claim, averaging of IDR 74,572,180 (USD 5178).^3^ Hospital tariff is set by the hospital for COVID-19 related services, which is different based on distinct types and classes of hospitals. Secondly, total claims or charges were submitted to be reimbursed by the hospital to the MOH system, following calculation as mentioned in the methodology.

Other payments such as top-ups and deductibles were also applied in the claim payment scheme, which serves to disincentivize for incomplete administrative and lateness of claims details. Top-ups and deductibles were part of the earlier reimbursement policy formula established by the Ministry of Health in 2021. As per technical guidance (Minister of Health’s Decree No. 5673 of 2021), the top-ups were calculated per utilization of certain additional services, which included the use of ventilator and/or plasma convalescent treatment. Deductibles employed a rather similar approach, where patients did not get tested for any laboratories or radiologic service. Subsequent thereafter, the reimbursement rate was deducted per test not being conducted.

Top-up averaging IDR 320,700 was made for additional services such as a cost of funeral due to COVID-19 related mortality. However, hospitals on average received deductibles from total submitted claims of IDR 1205,290, which was primarily due to the missing COVID-19 confirmation test.

There was a huge gap between the amount of tariff and charges for COVID-19 care, in which this gap creates a profit margin for hospitals to cover for COVID-19 related services. The huge gap was owing to the fact that ever since case-mix base group (CBG) tariff had not been adequately calculated for COVID-19 cases, hospitals charged patients much more than what they should pay. The average costs for COVID-19 treatments were merely $1276 and $1205 for public and private hospitals, respectively.

As evident in Table 4, the average total paid claim for COVID-19 case management in all severity was IDR 74,572,000 (USD 5178). Moreover, the hospital tariff had an average of IDR 30,098,590 (USD 2090) per patient, which consisted 40% of the average of total of submitted claims. There is indeed a difference between tariffs and claims which is expected to cover variance of tariffs between not only public and private hospitals, but also across different hospital classes which provide COVID-19 case management. This difference is particularly applicable as regards to private hospitals, which need to build infrastructure for managing COVID-19 patients. In the early days of the pandemic, hospitals needed PPEs (classified as consumables), an expensive-yet-required item in practice which at the start was charged to patients, leaving these patients to pay out-of-pocket despite regulations that were supposed to prevent this kind of charge.^4^ As the difference of the claims’ amount based on different proxies was observed, it is worth noting what predicts the amount of claim based on given data. It is particularly important to determine what indicators influence claims; thus, it serves as a basis of policy change for cost and quality control of COVID-19 care. We conducted weighted regression as to minimizing standard variance if simple regression is applied. The result of the regression table using the weighted approach is shown in Table 5.

**Table 4:** Average tariff and total claim charges

***N*** = 102.605		
Claim Component	Average^**a**^	SD
Hospital Claims	77,142.72	54,672.49
Top-up Funeral	320.70	941.62
Deductibles	1205.92	686.51
**Total Claim**	74,572.18	54,963.42
**Hospital Tariff**	30,098.59	38,797.3
*Difference Between Av. Claim and Tariffs*	44,473.6	45,313.26

^a^All in,000 IDR (thousand Rupiah)

**Table 5:** Weighted regression of multiple predictors to the claim amount using OLS

Predictors	β	SE	% Claim increase (per unit increase of predictor)
*Mild severity*	Ref		N/A
*Moderate severity*	0.00	0.01	0%
*Severe & critical cases*	0.12*	0.01	13%
*Class C*	Ref		N/A
*Class B*	0.02*	0.01	−5%
*Class A*	−0.04*	0,01	2%
*Non-ventilator use*	Ref		N/A
*Ventilator use*	0.53*	0,01	70%
*LOS*	0.09*	0,01	10%
*Inpatient & outpatient*	Ref		N/A
*ICU use*	0.52*	0.01	69%
*Recovered*	Ref		N/A
*Died*	0.09*	0.01	9%

*Significant at *p*-value 0.01

It was found that compared with mild severity, (per unit addition of-) severe and critical cases were associated with an increase in claim amount by 12% as shown in Table 5. The rationale was attributed to fact that severe cases implied more charges at the hospital level since procedures were applied and room occupancy often required intensive units, which led to the increase in claims.

Class of hospital despite its potential, was not a predictor of COVID-19 claims or payment amount as it gave mixed results in the analysis. Hospital class was found to be associated with both an increase and a decrease in claim amount; a unit addition of class B was associated with a 5% decrease while class A was associated with 2% increase. Such result suggests that respectively, claims in hospital class B were associated with only slightly lower charges while class A was only slightly higher. Despite the limited variance of charge among hospital class, the regression result informs the nuance of hospital standards, which was arguably affected by many factors, including different management patterns which are influenced by decentralization in Indonesia, as well as service standards. Furthermore, not all classes of hospital presented its ownership, whether it is publicly or privately owned, with the latter potentially absorbing more cost.

Treatment based on clinical indication was associated with an increasing claim amount, as shown in our study. (As per) Ventilator use, for example, was linked to 70% of claim amount increase. Moreover, an addition of ICU inpatient admission was associated with an increase of 69% claim amount. These findings were relevant since ventilator and ICU use was determined at different per-diem rate by the Decree of the MOH 446/2020 regulation on COVID-19 purchasing of secondary care. Moreover, (per addition of) morbid outcome (death) was associated with an augmentation of claim amount by 9%, which was attributed to additional costs for end-care treatment as well as such as funeral service, among others.

## Discussion

This is the first study that addresses COVID-19 claims payment system as well as its determinants using the recently established COVID-19 claims data in Indonesia. The variables used in the analysis were derived from the actual claims system, which informs the tariff, claims, as well as predictors of cost. Furthermore, this study adds to the breadth of evidence around the cost of COVID-19, as existing literature shows cost prediction using population-level data as well as economic proxies, the approach of which is different from other studies [8, 9]. Barasa et al. examined cost of management in Kenya, but by estimating the per-day unit cost from bottom approach, which is different from this study that used secondary data of claims [10].

The descriptive clinical pattern highlighted in this study is consistent with other studies [11–14]. In general, the mortality rate of COVID-19 patients found by this study is considerably lower than in a high-income country, especially at the early times of pandemic [11–14]. A recent study in the Indonesian context suggested that in-hospital mortality rate was 12%, while in this study it was around 13% [15].

The result of this study showed a considerable gap between average tariffs (averaging of IDR 30,098,590 or USD 2090) and actual claims (averaging of IDR 74,572,180 or USD 5178) submitted to the operator (MOH and national health insurance agency), which was and remains beneficial in supporting operational costs for managing COVID-19.^5^ It is possible to argue that this significant difference is expected to cover operational costs for managing COVID-19 besides clinical care. Infrastructure-wise, for example, hospitals need to build infrastructure for COVID-19 patients, including COVID-19 specific emergency room, COVID-19 inpatient isolation room, as well as operating rooms (when needed) [16]. According to WHO standards, a system needs to be put in place for infection prevention and control (IPC), such as supplies and regular environmental cleaning [17]. The trend brings about the need for incentives in managing COVID-19 cases [18, 19].

The result of the study sheds light on the predictor of COVID-19 claims and healthcare cost associated with the management of the disease. COVID-19 severity and the utilization of medical technologies correlate with a positive increase of the claims amount. This is consistent with studies by Naci et al. (2010) and Ivanova et al. (2012) that found the positive correlation between disease severity and healthcare cost [20, 21].

As mentioned within the claims regulation i.e., the Decree of MOH 446/2021 on Usage of Rapid Diagnostic Antigen Test in Examining Covid-19 test, usage of ventilator and ICU rooms increase the claims rate, thus, on the one hand, it is plausible that the increased claim amount is associated with the use of high-end medical technologies. On the other hand, variables such as the hospital class or hospital characteristics yielded mixed results. Vertical, class A hospital with public ownership tends to charge more for COVID-19 cases, unlike private class B hospital which is associated with less costs. Relevant to the case is the fact that class A hospital utilizes more resources as it has to provide specialized care. B class hospital on the other hand was spending less compared to class C or lower, despite having the higher authority and resources. It is argued that B class hospital, compared to class C, was dominated by public hospital, while class C was dominated more by private hospital, thus it would impact higher bills [22]. Moreover, before and during COVID-19, through public financing measure, public hospitals were receiving subsidy from public fund while private sectors were not, which impacts to lower cost.

In the long run, it is imperative to find the best estimate of cost as a benchmark for policymaking. Deriving results from claims or healthcare payment database patterns for informing utilization and clinical practice, or vice versa, has long been recorded in a myriad of studies. Medicaid uses its claims data to predict drivers of utilization of maternal care, including primary obstetric care as well as post-partum [23]. In a dynamic microsimulation model to predict the cost-effectiveness of Covid-19 public health intervention strategies in KwaZulu-Natal province, South Africa, the optimal combination of interventions was contingent on epidemic growth characteristics and efficacies and costs of interventions [24] .

Amidst the lack of standard protocols and growing clinical literature around COVID-19, there is a gap in linking the clinical and management practices of the disease and the actual cost of managing COVID-19 that needs to be urgently addressed. Evidence suggests there is a variance in COVID-19 management; clinical standards are warranted in some cases [25]. This variance in treatment affects cost, which would be burdensome, particularly in a number of countries with high incidence and risk of infection yet facing budget constraints. In the context of Indonesia, a retrospective payment system for COVID-19, despite accommodating COVID-19 payment to the hospitals, provides a little-to-no incentive for efficiency, potentially affecting the national budget.

Financing COVID-19 care through a retrospective fee-for-service payment system is unsustainable since it does not promote efficiency in the long run. The quest for seeking out the ideal payment model for COVID-19, hence, is justified. Several countries, including USA and the U.K, accommodate COVID-19 into their bundled payment systems under public health insurance scheme, either for managing COVID-19 cases in referral care or COVID-19 vaccine administrations [26, 27]. Accommodating care into alternative payment systems like bundled Diagnosis-Related Group (DRG) payment practice, we contend, promotes efficiency control and leverages quality of care as hospitals need to implement cost and quality control [28–30].

The COVID-19 payment system, while regarded as new, was established under a separate payment system from the national health insurance system. Bundled DRG payment as a secondary care payment system, or Indonesian Casemix-Based Groups (INACBGs) in Indonesia, has been implemented in Indonesia, particularly since the practice of the Indonesia national health insurance system (JKN). JKN covers both inpatient and outpatient services nationwide for almost 786 diagnosis groups of inpatient care [31]. Under the remit of payment arrangement, a hospital is paid a fixed price in managing a disease, which varies based on region and severity level of the disease. It is essential that COVID-19, despite the fact it was being established in a different payment scheme, necessitates for it to be integrated within INACBGs in the long run.

## Conclusion

This study highlights the average tariffs and charges for hospital COVID-19 care as predictor of cost of treatment for COVID-19 in Indonesia. This evidence serves pivotal as COVID-19 has become an integral part of the healthcare system. Thus, more efficient and effective payment policy needs to be made which ought to be drawn from evidence and findings of this study. Based on this study, we concluded that use of resources and disease severity is well correlated with amount of reimbursement. Hence, it is imperative to have a COVID-19 payment policy that is efficient in the long run.

The nature of COVID-19 which is rapidly evolving prompts for quick decision on policymaking, and hence, a rapid payment scheme was built. In the long term, as COVID-19 becomes more endemic, revision to healthcare payment policy especially for COVID-19 disease needs to be made; particularly one that is integrated with the existing payment scheme of the national health insurance. An example of this integration is Diagnosis Related Group (DRG) payment. Utilizing this payment scheme will lead to efficiency of payment and budget saving to the national budget without compromising quality of care.

## Data Availability

The data that support the findings of this study are available from the claims database of the Ministry of Health, Govt. of Indonesia, but restrictions apply to the availability of these data, which were used under license for the current study, and so are not publicly available. Data are however available from the authors upon reasonable request and with permission of Ministry of Health, Govt. of Indonesia.

## Abbreviations

COVID-19: Coronavirus Disease-19
GoI: Government of Indonesia
WHO: World Health Organization
MOH: Ministry of Health
SSAH: Social Security Agency for Health
PCR: Polymerase Chain Reaction
ICD: International Classification of Disease
ICU: Intensive Care Unit
LOS: Length of Stay
CT: Computed Tomography
IPC: Infection and Prevention Control
NHI: National Health Insurance
JKN: *Jaminan Kesehatan Nasional* (National Health Insurance, in *Bahasa)*
DRG: Diagnosis-Related Group
INACBGs: Indonesian Casemix Based Groups
UHC: Universal Health Coverage

## Footnotes

1 Calculation was made for referring to the policy made by the minister of health (i.e., the Minister of Health’s decree No. 413 Year 2020). The top-up is added based on the purchasing of plasma treatment as well as ventilators. The reimbursement for plasma treatment is determined by the Indonesia Red Cross at the unit cost of US$155 per plasma bag which has the same conversion rate with other rates applicable in Indonesia. For the funeral service, this refers to the same regulation which oversees the total of US$ 231 per deceased patient.

2 Person-under-surveillance (*Orang Dalam Pengawasan*/ODP) is suspected cases found after contact tracing, in which the person is allowed to self-isolate while waiting for confirmation of test result.

3 Calculated using rate USD 1 = IDR 14,400.

4 The regulation is based on the bylaw of 6/2018 on health quarantine, which states the coverage of service of COVID-19, including consumables such as PPE, is covered by the government. This is later translated into claims coverage technical guideline (Decree MOH 446/2020) which regulates coverage of PPE in hospitals.

5 Calculated using rate USD 1 = IDR 14,400.

## References

[1] Worldometers (2021). Indonesia COVID-19 cases update.

[2] Brosi D (2021). Local public health system capacity and its relationship to COVID-19 mortality patterns. Health Serv Res.

[3] GoI DG of HSM of H (2021). COVID-19 health service update.

[4] Mboi N (2015). Indonesia: on the way to universal health care. Heal Syst Reform.

[5] Ministry of Health Republic of Indonesia (2020). Technical guidance on claim payment for COVID-19 cases. HK.01.07/MENKES/446/2020.

[6] Kementerian Kesehatan Republik Indonesia (2021). The Decree of the Minister of Health 4344/2021 on technical guidance of claim payment for COVID-19 management reimbusement.

[7] Pattnaik A, Nugraha RR, Thabrany H, Connor C (2020). Lessons from Rapid Implementation: how the revamped system to purchase COVID services in Indonesia affected its hospitals.

[8] Jin H, Wang H, Li X, Zheng W, Ye S, Zhang S (2020). Economic burden of COVID-19, China, January-March, 2020: a cost-of-illness study. Bull World Health Organ.

[9] Czernichow S, Bain SC, Capehorn M, Bøgelund M, Madsen ME, Yssing C (2021). Costs of the COVID-19 pandemic associated with obesity in Europe: a health-care cost model. Clin Obes.

[10] Barasa E, Kairu A, Ng’Ang’A W, Maritim M, Were V, Akech S (2021). Examining unit costs for COVID-19 case management in Kenya. BMJ Glob Heal.

[11] Giacomelli A, Ridolfo AL, Milazzo L, Oreni L, Bernacchia D, Siano M (2020). 30-day mortality in patients hospitalized with COVID-19 during the first wave of the Italian epidemic: a prospective cohort study. Pharmacol Res.

[12] Docherty AB, Harrison EM, Green CA, Hardwick HE, Pius R, Norman L (2020). Features of 20 133 UK patients in hospital with covid-19 using the ISARIC WHO Clinical Characterisation Protocol: prospective observational cohort study. BMJ.

[13] Lewnard JA, Liu VX, Jackson ML, Schmidt MA, Jewell BL, Flores JP, et al. Incidence, clinical outcomes, and transmission dynamics of severe coronavirus disease 2019 in California and Washington: prospective cohort study. BMJ. 2020;369(9). 10.1136/bmj.m1923 PMC7243800 32444358

[14] Macedo A, Gonçalves N, Febra C (2021). COVID-19 fatality rates in hospitalized patients: systematic review and meta-analysis. Ann Epidemiol.

[15] Surendra H, Elyazar IR, Djaafara BA, Ekawati LL, Saraswati K, Adrian V (2021). Clinical characteristics and mortality associated with COVID-19 in Jakarta, Indonesia: a hospital-based retrospective cohort study. Lancet Reg Heal - West Pacific.

[16] Ministry of Health Republic of Indonesia (2020). Technical guidance on health services during the COVID-19 adaptation period.

[17] World Health Organization. Infection prevention and control health-care facility response for COVID-19. World Heal Organ. 2020;(October):32 Available from: https://www.who.int/publications/i/item/WHO-2019-nCoV-HCF_assessment-IPC-2020.1.

[18] Giannouchos TV, Biskupiak J, Moss MJ, Brixner D, Andreyeva E, Ukert B (2021). Trends in outpatient emergency department visits during the COVID-19 pandemic at a large, urban, academic hospital system. Am J Emerg Med.

[19] Jeffery MM, D’Onofrio G, Paek H, Platts-Mills TF, Soares WE, Hoppe JA (2020). Trends in emergency department visits and hospital admissions in health care systems in 5 states in the first months of the COVID-19 pandemic in the US. JAMA Intern Med.

[20] Naci H, Fleurence R, Birt J, Duhig A (2010). Economic burden of multiple sclerosis. Pharmacoeconomics.

[21] Ivanova JI, Bergman R, Birnbaum HG, Colice GL, Silverman RA, McLaurin K (2012). Effect of asthma exacerbations on health care costs among asthmatic patients with moderate and severe persistent asthma. J Allergy Clin Immunol.

[22] Soewondo P, Mahendradhata Y, Listyadewi S, Marthias T, Harimurti P, Prawira J (2017). The Republic of Indonesia health system review.

[23] Bennett WL, Chang H-Y, Levine DM, Wang L, Neale D, Werner EF (2014). Utilization of primary and obstetric care after medically complicated pregnancies: an analysis of medical claims data. J Gen Intern Med.

[24] Reddy KP, Shebl FM, Foote JHA, Harling G, Scott JA, Panella C (2021). Cost-effectiveness of public health strategies for COVID-19 epidemic control in South Africa: a microsimulation modelling study. Lancet Glob Heal.

[25] Azoulay E, de Waele J, Ferrer R, Staudinger T, Borkowska M, Povoa P (2020). International variation in the management of severe COVID-19 patients. Crit Care.

[26] Center for Medicare and Medicaid Services (CMS) (2021). Medicare COVID-19 vaccine shot payment.

[27] The UK National Health Services (NHS) (2021). Frequently asked questions on financial response to COVID-19.

[28] Agarwal R, Liao JM, Gupta A, Navathe AS (2020). The impact of bundled payment on health care spending, utilization, and quality: a systematic review. Health Aff.

[29] Struijs J, De Vries EF, Baan CA, Van Gils PF, Rosenthal MB (2020). Bundled-payment models around the world: how they work and what their impact has been. Abstract.

[30] Adida E, Mamani H, Nassiri S (2016). Bundled payment vs. fee-for-service: impact of payment scheme on performance. Manage Sci.

[31] Agustina R, Dartanto T, Sitompul R, Susiloretni KA, Suparmi, Achadi EL (2019). Universal health coverage in Indonesia: concept, progress, and challenges. Lancet.

[32] Gebhard C, Regitz-Zagrosek V, Neuhauser HK, Morgan R, Klein SL (2020). Impact of sex and gender on COVID-19 outcomes in Europe. Biol Sex Differ.

